# Development and verification of a nomogram for predicting short-term mortality in elderly ischemic stroke populations

**DOI:** 10.1038/s41598-023-39781-4

**Published:** 2023-08-03

**Authors:** Guangyong Jin, Wei Hu, Longhuan Zeng, Mengyuan Diao, Hui Chen, Jiayi Chen, Nanyuan Gu, Kai Qiu, Huayao Lv, Lu Pan, Shaosong Xi, Menglu Zhou, Dongcheng Liang, Buqing Ma

**Affiliations:** 1 Department of Critical Care Medicine, Affiliated Hangzhou First People’s Hospital, Zhejiang University School of Medicine, Hangzhou, China; 2 Department of Intensive Care Unit, The Affiliated Hospital of Hangzhou Normal University, Hangzhou, China; 3 Department of Neurology, The Affiliated Hospital of Hangzhou Normal University, Hangzhou, China

**Keywords:** Stroke, Outcomes research, Stroke, Stroke

## Abstract

Stroke is a major healthcare problem worldwide, particularly in the elderly population. Despite limited research on the development of prediction models for mortality in elderly individuals with ischemic stroke, our study aimed to address this knowledge gap. By leveraging data from the Medical Information Mart for Intensive Care IV database, we collected comprehensive raw data pertaining to elderly patients diagnosed with ischemic stroke. Through meticulous screening of clinical variables associated with 28-day mortality, we successfully established a robust nomogram. To assess the performance and clinical utility of our nomogram, various statistical analyses were conducted, including the concordance index, integrated discrimination improvement (IDI), net reclassification index (NRI), calibration curves and decision curve analysis (DCA). Our study comprised a total of 1259 individuals, who were further divided into training (n = 894) and validation (n = 365) cohorts. By identifying several common clinical features, we developed a nomogram that exhibited a concordance index of 0.809 in the training dataset. Notably, our findings demonstrated positive improvements in predictive performance through the IDI and NRI analyses in both cohorts. Furthermore, calibration curves indicated favorable agreement between the predicted and actual incidence of mortality (P > 0.05). DCA curves highlighted the substantial net clinical benefit of our nomogram compared to existing scoring systems used in routine clinical practice. In conclusion, our study successfully constructed and validated a prognostic nomogram, which enables accurate short-term mortality prediction in elderly individuals with ischemic stroke.

## Introduction

Stroke poses a substantial and escalating healthcare challenge worldwide, particularly in the elderly segment of the population. Projections suggest that by 2050, the number of stroke survivors will surpass 200 million, with 25 million new stroke cases and 13 million stroke-related deaths occurring annually^1^. Ischemic stroke, a pervasive and devastating subtype of stroke, significantly contributes to disability and mortality, thereby imposing a considerable burden on patients, their families, society, and nations at large^2–4^. The aging population is a crucial factor driving the mounting burden of stroke worldwide^1^. With ongoing demographic shifts, the prevalence of elderly individuals affected by ischemic stroke is expected to rise significantly. The burden of ischemic stroke in the elderly is substantial, and mortality rates are notably higher among this population^5^. Therefore, the identification of prognostic factors and the ability to discern elderly ischemic stroke patients at a heightened risk of adverse outcomes have become pressing priorities.

Nomograms have emerged as a valuable visual tool for predicting survival outcomes in ischemic stroke patients. While we have previously developed a user-friendly nomogram that effectively predicts long-term mortality in ischemic stroke patients^6^, there is a scarcity of nomograms specifically designed to predict short-term mortality in this population. An attempt to develop a nomogram utilizing the triglyceride-to-high-density lipoprotein cholesterol ratio for predicting 3-month mortality yielded a disappointing concordance index (C-index) of 0.684, indicating suboptimal predictive accuracy^7^. Regrettably, there is a paucity of studies focusing on the development of nomograms to predict clinical outcomes in elderly patients with ischemic stroke. Notably, a nomogram has been established in the Chinese population to predict the 6-year incidence rate of stroke among middle-aged and elderly individuals^8^, while another nomogram has been employed to personalize the prediction of adverse outcomes in elderly patients following mechanical thrombectomy for acute stroke^9^. To our knowledge, the development of nomograms for predicting short-term mortality in elderly ischemic stroke patients is currently lacking. hold significant potential for comprehensive mortality evaluation, enhancing communication of medical conditions, and ultimately mitigating the occurrence of medical disputes^10^.

Our objective was to integrate a multitude of independent risk factors and develop a dedicated nomogram capable of predicting 28-day mortality in elderly patients with ischemic stroke. To accomplish this, we utilized readily available clinical data from the Medical Information Mart for Intensive Care IV (MIMIC-IV) database.

## Methods

### Data source

The entirety of our raw data was sourced from version 2.1 of the MIMIC-IV database. This extensive database encompasses the medical records of over 70,000 individuals who were admitted to the intensive care unit (ICU) at Beth Israel Deaconess Medical Center (BIDMC) in Boston, Massachusetts, spanning the period from 2008 to 2019. The dataset comprised various documented clinical parameters, including vital signs, laboratory measurements, medication details, and more^11^. Ethical approval for the establishment of the MIMIC-IV database was obtained from the institutional review boards at Massachusetts Institute of Technology (MIT) and BIDMC. One of the authors, GJ, holds PhysioNet credentials (https://www.physionet.org) and has successfully completed training in human subject research (Certification number: 46141344). Given the retrospective nature of our study, informed consent requirements were waived in compliance with national regulations and agency guidelines.

### Study subjects and data extraction

**Figure 1:**
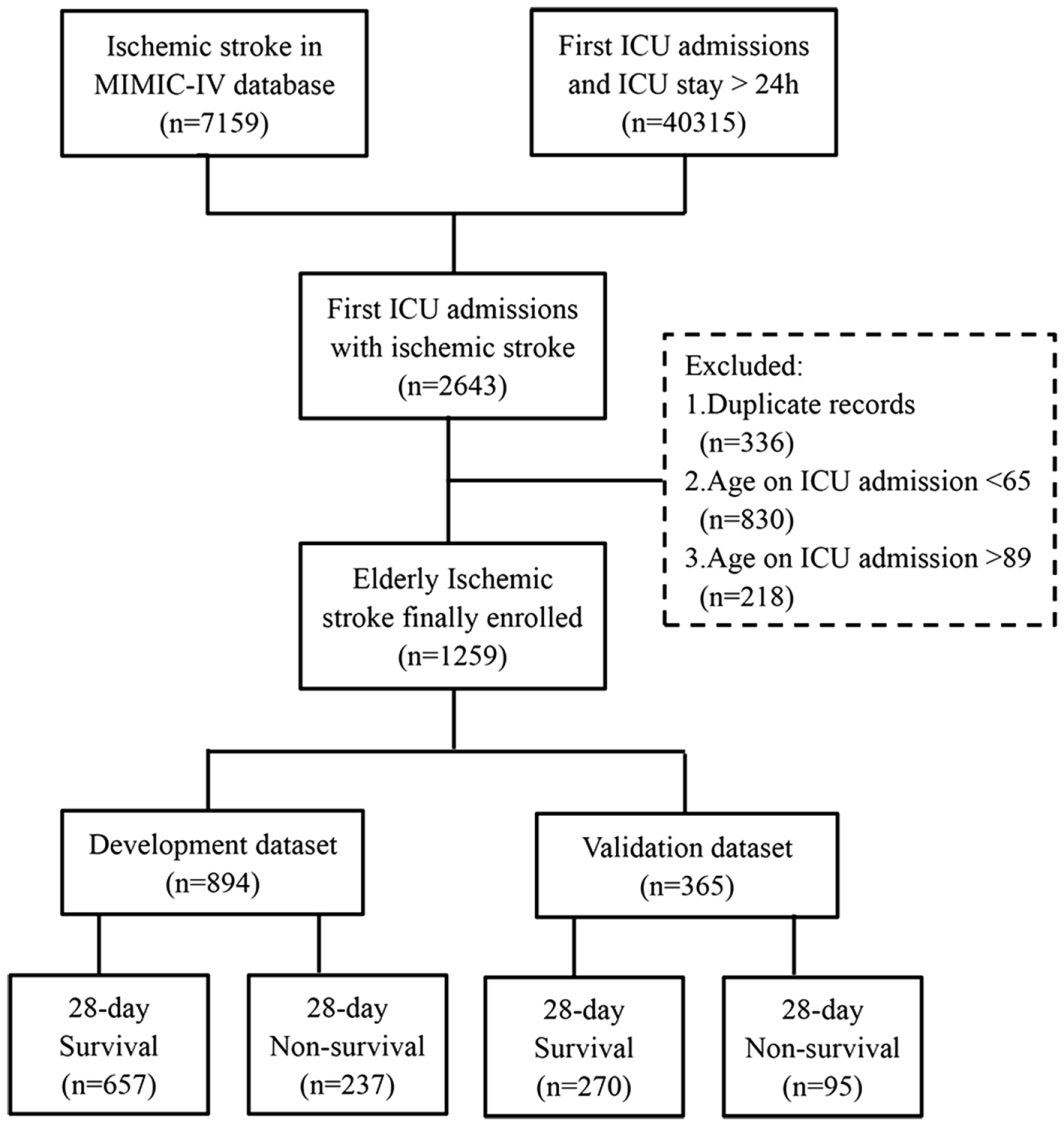
Flowchart for Study Subject Selection. *MIMIC-IV* medical information mart for intensive care IV, *ICU* intensive care unit.

Our study focused on recruiting elderly individuals with ischemic stroke who were admitted to the ICU for the first time. To ensure data consistency, we excluded information related to subsequent ICU stays (2nd and onwards). Ischemic stroke cases were identified following the guidelines provided by the International Classification of Diseases (ICD), encompassing both version 9 and version 10, as previously established^12^. To enhance the homogeneity of our study population, we further excluded duplicate records during the initial ICU stay, records of non-elderly patients (under 65 years old), and records of very elderly patients (over 89 years old). Moreover, in order to minimize variability, subjects with an ICU length of stay (LOS) of less than one day were excluded from the analysis. Ultimately, a total of 1259 elderly patients with ischemic stroke were identified and randomly divided into development and validation cohorts in a 7:3 ratio, with the seed set at 17 (Fig. 1).

The following variables were extracted systematically: age, gender, weight, marital status, ethnicity, first care unit, hospital/ICU admission time, hospital/ICU discharge time, death time (if applicable), hospital LOS, and ICU LOS. MIMIC-IV concepts were generated, serving as useful abstractions of the raw data. Specifically, we retained the Acute Physiology Score III (APS III), Simplified Acute Physiology Score II (SAPS II), Sequential Organ Failure Assessment (SOFA), Oxford Acute Severity of Illness Score (OASIS), Logistic Organ Dysfunction System (LODS), Glasgow Coma Scale (GCS), vital signs, biochemical indicators, and blood routine examination results from the first day of ICU hospitalization, all based on the concept framework provided by the database. Furthermore, comorbidities and Charlson comorbidity index (CCI) were identified using the Charlson table derived from the MIMIC-IV concepts^13^, in accordance with a previously conducted study^14^. Additionally, we ascertained the usage of various treatments during the initial day of ICU admission, including mechanical ventilation (MV), antiplatelet agents, heparin, alteplase, albumin, furosemide, mannitol, and vasopressors. To capture the temporal dynamics of these variables, we extracted data collected multiple times throughout the first day of ICU hospitalization using the MIMIC concepts. For each variable of interest, the minimum, maximum, and average values (if available) were utilized for subsequent analyses.

### Statistical analysis

To identify outliers, we utilized histograms and applied winsorization using the winsor2 command in Stata software, replacing extreme values beyond the 0.5% and 99.5% thresholds. To address missing values, multiple imputations were performed. Random allocation of individuals to either the development or validation datasets was conducted using R software. Statistical methods were employed to assess differences between the training and validation cohorts, including: (1) Normality tests such as skewness and kurtosis tests for continuous variables. (2) In cases where continuous variables did not exhibit normal distribution, the Mann–Whitney U-test was employed, and median and interquartile range (IQR) values were reported. (3) Categorical variables were presented as percentages and assessed using the Chi-square test.

Subsequent analyses were conducted in both Stata software (version 17.0, Stata Corporation LLC, College Station, USA) and R software (version 4.2.1, R Foundation for Statistical Computing, Vienna, Austria), utilizing various packages including rms, car, glmnet, pROC, regplot, PredictABEL, and rmda data packages. To filter out relevant variables from the extracted data in the training set, a tenfold cross-validation procedure and the least absolute shrinkage and selection operator (LASSO) regression were employed, as previously described^15^. Binary logistic regression was then performed with the 28-day survival state as the dependent variable, leading to the construction of a nomogram for predicting 28-day mortality in elderly ischemic stroke patients following ICU admission. The performance of the nomogram was compared to other logistic regression models based on the SOFA, SAPS II, LODS, APS III and OASIS. Discrimination of the various models in both datasets was evaluated using the C-index, with values greater than 0.7 considered indicative of good discrimination^16^. To assess improvements in predictive performance, integrated discrimination improvement (IDI) and the net reclassification index (NRI) were calculated^17^. Calibration curves were generated using the 'val.prob' function to evaluate the accuracy of the models in both datasets. Additionally, decision curve analysis (DCA) was conducted on the training set to assess the clinical benefits and utility of the models. The significance level for statistical tests was set at *P* < 0.05.

### Ethics statement

The human participants involved in this study were reviewed and approved by Massachusetts Institute of Technology (MIT, Cambridge, MA) and Beth Israel Deaconess Medical Center (BIDMC, Boston, MA). This study was reviewed and approved by the institutional review board of Hangzhou First People’s Hospital. In order to protect the privacy of patients, the data was de-identified. Therefore, informed consent was abandoned by the institutional review boards of MIT and BIDMC. The authors confirm that all methods were carried out in accordance with relevant guidelines and regulations.

## Results

### Baseline characteristics

Table 1 presents an overview of the baseline characteristics of the development and validation datasets used in this study. The development set consisted of 894 elderly individuals with ischemic stroke, with a median age of 77.00 years (interquartile range [IQR]: 71.13, 82.67), and a female proportion of 52.01%. Similarly, the validation set included 365 elderly patients with ischemic stroke, with a median age of 76.30 years (IQR: 70.12, 83.14), and a female proportion of 53.15%. Importantly, no statistically significant differences were observed between the development and validation cohorts across all characteristics (*P* > 0.05), indicating reasonable grouping of all elderly ischemic stroke patients.

**Table 1:** Baseline characteristics of enrolled geriatric ischemic stroke patients.

Characteristics	All patients (n = 1259)	Development dataset (n = 894)	Validation dataset (n = 365)	*P-*value
Age, median (IQR)	76.83 (70.93, 82.82)	77.00 (71.13, 82.67)	76.30 (70.12, 83.14)	0.732
Female, No. (%)	659 (52.34)	465 (52.01)	194 (53.15)	0.714
Weight, median (IQR) (kg)	76.00 (64.30, 89.20)	76.30 (65.00, 88.50)	75.30 (63.60, 90.60)	0.963
Marital status, No. (%)				
Married	551 (43.76)	397 (44.41)	154 (42.19)	0.385
Single	227 (18.03)	166 (18.57)	61 (16.71)	
Other	481 (38.20)	331 (37.02)	150 (41.10)	
First care unit, No. (%)				
Medical ICU	112 (8.90)	74 (8.28)	38 (10.41)	0.325
Surgical ICU	396 (31.45)	284 (31.77)	112 (30.68)	
Medical ICU/Surgical ICU	59 (4.69)	41 (4.59)	18 (4.93)	
Neuro surgical ICU	172 (13.66)	113 (12.64)	59 (16.16)	
Trauma surgical ICU	128 (10.17)	97 (10.85)	31 (8.49)	
Other ICU	392 (31.14)	285 (31.88)	107 (29.32)	
Underlying diseases, No. (%)				
Myocardial infarct	225 (17.87)	151 (16.89)	74 (20.27)	0.155
Congestive heart failure	339 (26.93)	237 (26.51)	102 (27.95)	0.602
Chronic pulmonary disease	251 (19.94)	178 (19.91)	73 (20.00)	0.971
Rheumatic disease	45 (3.57)	34 (3.80)	11 (3.01)	0.494
Peptic ulcer disease	23 (1.83)	15 (1.68)	8 (2.19)	0.537
Liver disease	51 (4.05)	39 (4.36)	12 (3.29)	0.380
Renal disease	237 (18.82)	173 (19.35)	64 (17.53)	0.454
Diabetes	468 (37.17)	338 (37.81)	130 (35.62)	0.465
Paraplegia	627 (49.80)	443 (49.55)	184 (50.41)	0.782
Metastatic solid tumor	44 (3.49)	29 (3.24)	15 (4.11)	0.448
CCI, median (IQR)	8.00 (6.00, 9.00)	8.00 (6.00, 9.00)	8.00 (7.00, 9.00)	0.582
Disease severity score, median (IQR)				
Firstday SOFA	4.00 (2.00, 6.00)	4.00 (2.00, 6.00)	4.00 (2.00, 6.00)	0.986
Firstday LODS	4.00 (2.00, 7.00)	4.00 (2.00, 7.00)	4.00 (2.00, 7.00)	0.725
Firstday OASIS	34.00 (28.00, 41.00)	34.00 (28.00, 41.00)	34.00 (28.00, 41.00)	0.772
Firstday APS III	44.00 (32.00, 61.00)	43.00 (32.00, 60.00)	45.00 (32.00, 62.00)	0.809
Firstday SAPS II	35.00 (29.00, 44.00)	35.00 (29.00, 44.00)	36.00 (29.00, 43.00)	0.788
Vital indicators, median (IQR)				
Temperature (°C)**	36.88 (36.66, 37.13)	36.89 (36.67, 37.14)	36.85 (36.66, 37.12)	0.396
Heart rate (beats/min)**	79.64 (70.15, 90.11)	79.84 (70.27, 90.52)	78.26 (69.92, 88.77)	0.399
Respiratory rate (breaths/min)**	18.79 (16.87, 20.93)	18.80 (16.96, 20.86)	18.76 (16.79, 21.25)	0.873
SBP (mmHg)**	131.87 (118.88, 145.22)	132.42 (119.50, 145.27)	130.47 (117.70, 145.03)	0.368
Oxygen saturation (%)**	97.23 (95.92, 98.50)	97.24 (95.85, 98.50)	97.23 (96.12, 98.48)	0.543
Firstday GCS*	11.00 (8.00, 14.00)	11.00 (8.00, 14.00)	12.00 (8.00, 14.00)	0.784
Glucose (mmol/L)**	7.30 (6.15, 8.97)	7.25 (6.11, 8.84)	7.43 (6.27, 9.32)	0.185
Firstday urine output (L)	1.44 (0.94, 2.11)	1.46 (0.95, 2.10)	1.40 (0.93, 2.13)	0.715
Laboratory indicators, median (IQR)				
White blood cells (K/uL)***	11.40 (8.70, 15.40)	11.20 (8.60, 15.30)	11.70 (9.10, 15.40)	0.179
Platelets (K/uL)***	215.00 (169.00, 272.00)	213.50 (172.00, 269.00)	217.00 (164.00, 276.00)	0.928
Blood urea nitrogen (mg/dL)***	7.50 (5.36, 10.35)	7.44 (5.36, 10.35)	7.50 (5.71, 10.35)	0.477
Creatinine (μmmol/L)***	88.40 (70.72, 123.76)	88.40 (70.72, 123.76)	88.40 (70.72, 114.92)	0.588
Sodium (mEq/L)***	140.60 (138.00, 143.00)	141.00 (138.00, 143.00)	140.00 (138.00, 143.00)	0.233
Potassium (mEq/L)***	4.30 (3.90, 4.70)	4.30 (3.90, 4.70)	4.30 (3.90, 4.70)	0.649
Prothrombin time (sec)***	13.30 (12.00, 15.50)	13.30 (12.00, 15.50)	13.30 (11.90, 15.50)	0.887
International normalized ratio***	1.20 (1.10, 1.40)	1.20 (1.10, 1.40)	1.20 (1.10, 1.40)	0.869
Anion gap (mmol/L)***	16.00 (14.00, 18.00)	16.00 (14.00, 18.00)	16.00 (14.00, 18.00)	0.858
Intervention measures, No. (%)				
Endovascular obstruction removal	118 (9.37)	78 (8.72)	40 (10.96)	0.217
Alteplase	25 (1.99)	15 (1.68)	10 (2.74)	0.220
Antiplatelet	338 (26.85)	245 (27.40)	93 (25.48)	0.484
Heparin	375 (29.79)	268 (29.98)	107 (29.32)	0.816
Mannitol	34 (2.70)	24 (2.68)	10 (2.74)	0.956
Vasoactive agent	302 (23.99)	212 (23.71)	90 (24.66)	0.722
Invasive mechanical ventilation	436 (34.63)	305 (34.12)	131 (35.89)	0.548
Intracranial pressure monitor	34 (2.70)	26 (2.91)	8 (2.19)	0.477
Outcomes				
28-Day mortality (%)	332 (26.37)	237 (26.51)	95 (26.03)	0.860
ICU mortality (%)	186 (14.77)	130 (14.54)	56 (15.34)	0.716
Hospital mortality (%)	267 (21.21)	186 (20.81)	81 (22.19)	0.585
ICU LOS (days)	3.31 (1.94, 6.98)	3.21 (1.92, 6.88)	3.61 (2.00, 7.31)	0.423
Hospital LOS (days)	8.53 (4.93, 16.70)	8.74 (4.94, 16.65)	8.29 (4.93, 16.81)	0.883

The term "antiplatelet" was operationally defined as the administration of aspirin, clopidogrel, or dipyridamole within the first 24 h following admission to the ICU. Similarly, the term "vasoactive agent" was operationally defined as the administration of norepinephrine, epinephrine, phenylephrine, dopamine, dobutamine, vasopressin, or milrinone within the first 24 h following ICU admission. Within this same timeframe, assessments were conducted on disease severity scores, vital indicators, laboratory indicators, and interventions.

*CCI* Charlson Comorbidity Index, *ICU* Intensive Care Unit, *IQR* Interquartile Range; *GCS* Glasgow Coma Scale, *APS III* Acute Physiology Score III, *SOFA* Sequential Organ Failure Assessment, *LODS* Logistic Organ Dysfunction System, *SAPS II* Simplified Acute Physiology Score II, *OASIS* Oxford Acute Severity of Illness Score, *SBP* Systolic Blood Pressure, *LOS* Length of Stay.

*The minimum value of indicators on the firstday of ICU stay.

**The mean value of indicators on the firstday of ICU stay.

***The maximum value of indicators on the firstday of ICU stay.

### Variable selection and nomogram construction

**Figure 2:**
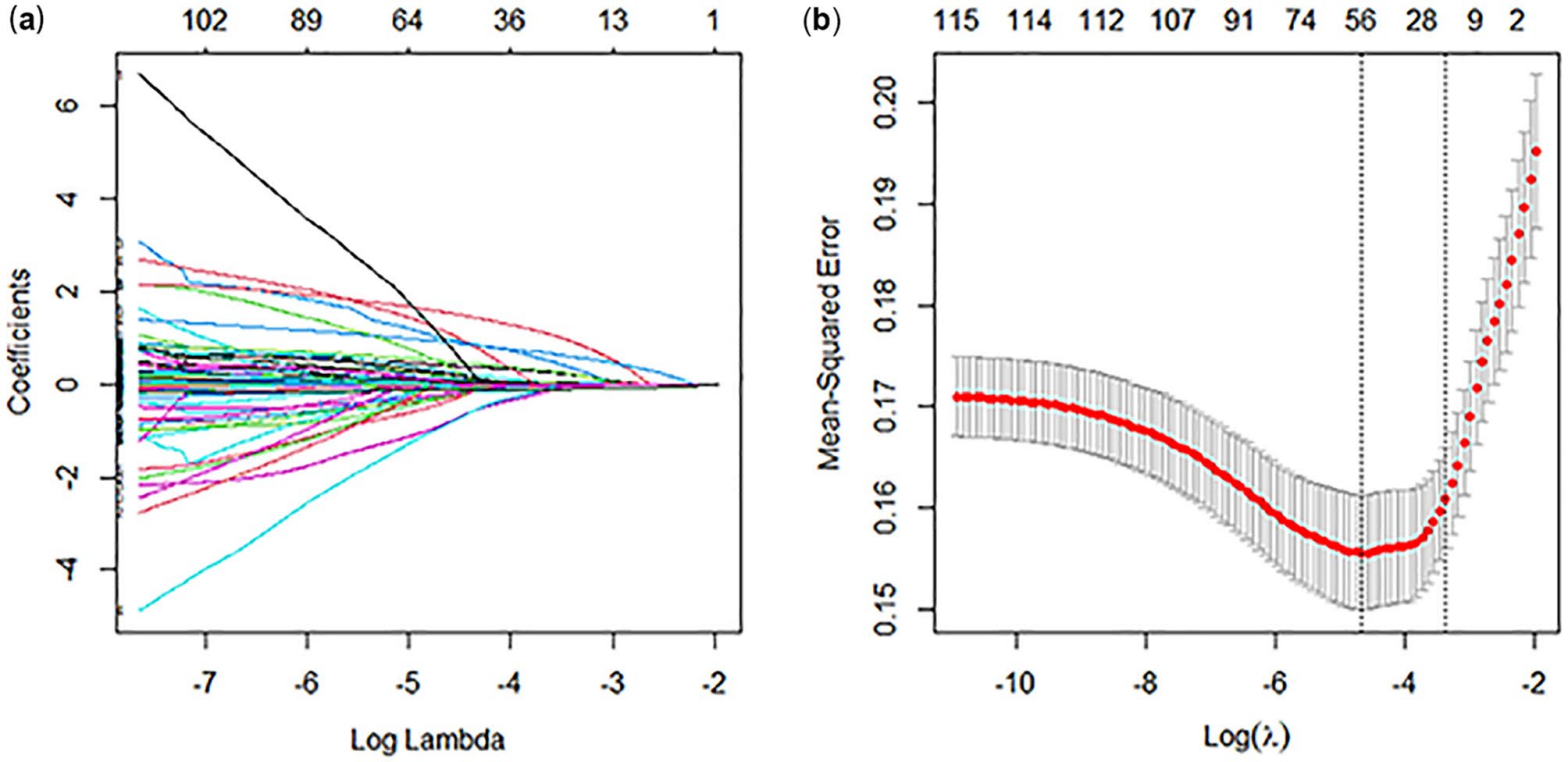
Feature selection process conducted through LASSO regression and tenfold cross-validation. (**a**) The figure illustrates the relationship between the coefficients of clinical features and the lambda value in the plot. As the lambda increased, the coefficients for each feature converged towards zero, indicating the regularization effect inherent in the LASSO regression. This regularization process effectively helps in identifying and selecting relevant features while mitigating potential overfitting in the model; (**b**) The tenfold cross-validation curve for LASSO regression is depicted, offering valuable insights into model selection. On the plot, the left dotted vertical line corresponds to the number of features and the optimal log (lambda) value that yielded the smallest mean squared error (λ = 0.009451193). Moreover, utilizing the one standard error criteria of the optimal log (lambda), the right dotted vertical line represents the model with 19 variables, striking a harmonious balance between predictive accuracy and model simplicity (λ = 0.03476508). This thoughtful selection of variables ensures robust performance while avoiding unnecessary complexity in the predictive model. *λ* lambda.

**Figure 3:**
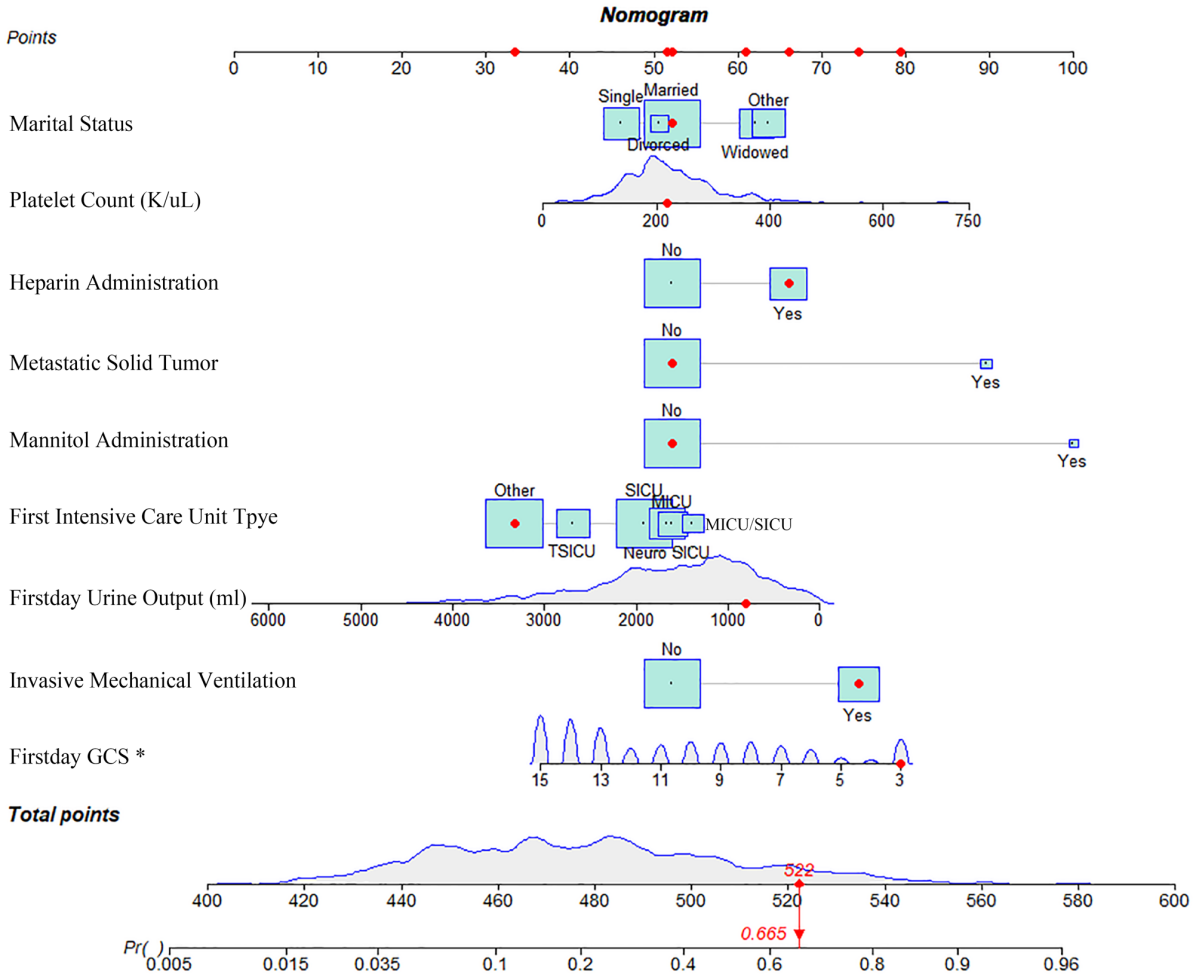
Nomogram for predicting 28-day mortality among elderly ischemic stroke patients. A red dot on the nomogram represents a specific patient's characteristics. In this example, the patient is married, without a history of metastatic solid tumor, and was admitted to the ICU of the "other" type. The patient's first-day urine volume was 800ml, GCS score was 3 points, and the maximum platelet count recorded was 218K/uL. The patient did not receive mannitol, but accepted heparin anticoagulation and mechanical ventilation. The sum of these specific points, calculated as 522, corresponds to a location on the total points line. From this point, a solid red line is drawn vertically down to the survival axis, indicating a risk probability of 28-day mortality, which in this case is 66.5%. This nomogram serves as a valuable clinical tool, allowing healthcare professionals to estimate individual patient risk and make informed decisions regarding patient care.

The selection of clinical features was performed using LASSO regression, and the results are presented in Fig. 2. A total of 9 independent variables demonstrated associations with 28-day mortality following initial ICU admission (*P* < 0.05). These variables included marital status (single, widowed, divorced, other), type of first care unit (Medical Intensive Care Unit [MICU], Surgical Intensive Care Unit [SICU], MICU/SICU, Neuro Surgical Intensive Care Unit [Neuro SICU], Trauma Surgical Intensive Care Unit [TSICU], Other), presence of metastatic solid tumor (yes), first-day urine output (L), platelet count (K/uL), mannitol administration (Yes), heparin administration (Yes), MV (Yes), and minimum value of first-day GCS. These significant factors were utilized in the construction of the nomogram, as illustrated in Fig. 3. Detailed information regarding the associations of these variables with 28-day mortality can be found in Table 2.

**Table 2:** Identification of independent risk factors for 28-day mortality among elderly ischemic stroke patients using multivariable logistic regression: insights from the development dataset.

Variables	OR	95% CI	*P*-value
Marital status (Single)	0.756	0.447–1.257	0.288
Marital status (Widowed)	1.584	0.981–2.546	0.058
Marital status (Divorced)	0.931	0.388–2.114	0.868
Marital status (Other)	1.691	1.025–2.778	0.039
First careunit (SICU)	0.857	0.465–1.593	0.623
First careunit (MICU/SICU)	1.120	0.422–2.896	0.817
First careunit (Neuro SICU)	0.969	0.477–1.970	0.930
First careunit (TSICU)	0.582	0.271–1.236	0.161
First careunit (Other)	0.421	0.221–0.802	0.008
Metastatic solid tumor (Yes)	5.569	2.228–14.191	< 0.001
Firstday urineoutput (L)	0.999	0.999–1.000	< 0.001
Platelet count (K/uL)***	1.003	1.001–1.005	< 0.001
Mannitol administration (Yes)	8.980	3.557–24.988	< 0.001
Heparin administration (Yes)	1.887	1.293–2.757	0.001
Mechanical ventilation (Yes)	2.766	1.915–4.008	< 0.001
Firstday GCS*	0.848	0.808–0.890	< 0.001

*OR* odd ratio, *CI* confidence interval, *GCS* Glasgow Coma Scale, *SICU* Surgical Intensive Care Unit, *MICU* Medical Intensive Care Unit, *Neuro SICU* Neuro Surgical Intensive Care Unit, *TSICU* Trauma Surgical Intensive Care Unit.

*The minimum value of indicators on the firstday of ICU stay.

***The maximum value of indicators on the firstday of ICU stay.

### Nomogram evaluation and validation

**Figure 4:**
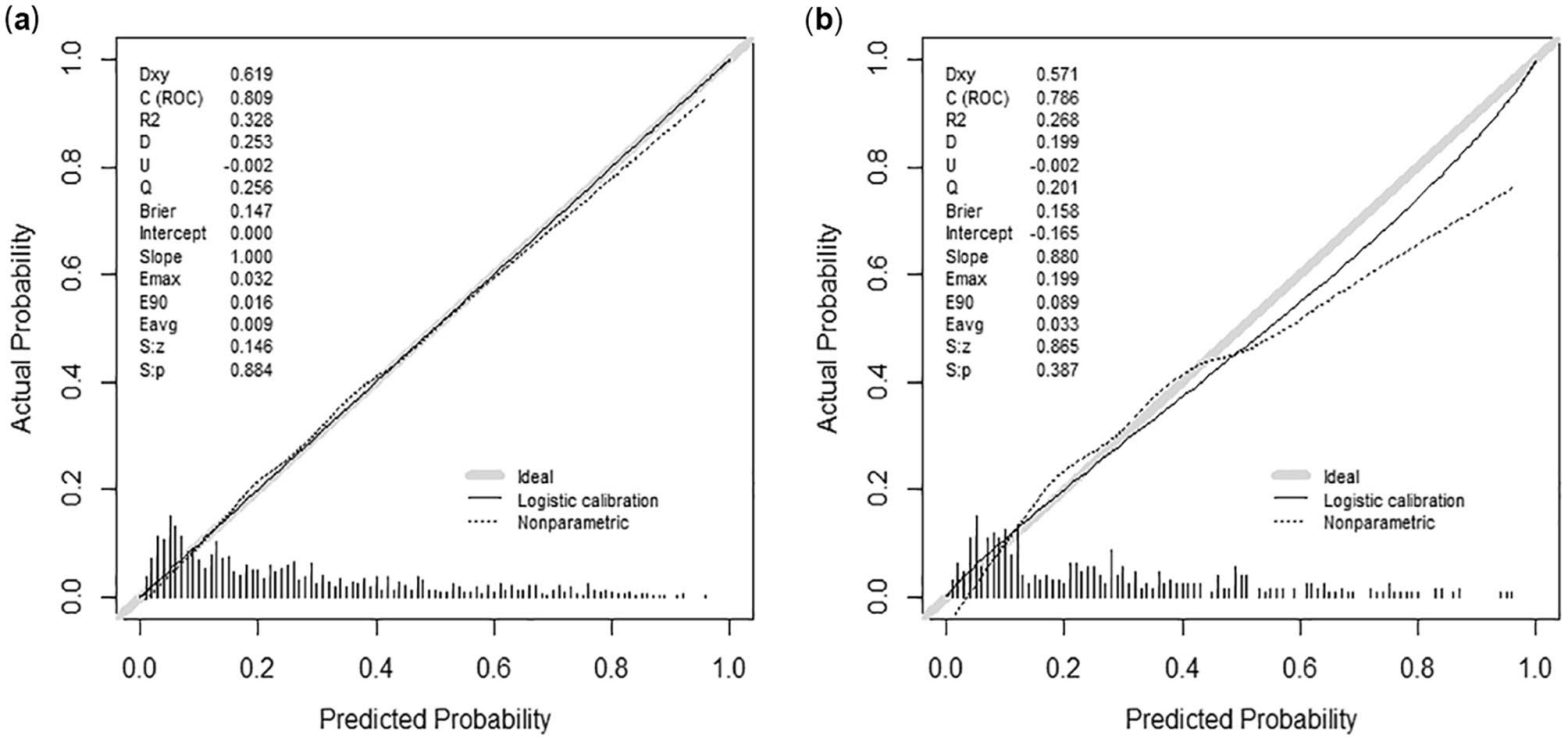
The calibration curve of the developed nomogram revealed a satisfactory alignment between the predicted and observed 28-day mortality in both the development (**a**) and validation (**b**) sets (All *P* > 0.05).

In the development dataset, our nomogram exhibited a C-index of 0.809 (95% CI 0.778, 0.841), indicating its strong accuracy in predicting the prognosis of elderly individuals with ischemic stroke. A similar C-index of 0.786 (95% CI 0.737, 0.835) was observed in the validation set, further supporting the robust performance of our nomogram. Notably, our nomogram outperformed the commonly used clinical scoring systems, including SOFA, APS III, LODS, SAPS II, and OASIS scores, as indicated by the higher C-index values observed in both datasets (Table 3). The IDI and NRI analyses further validated the superior predictive performance of our nomogram compared to the aforementioned scoring systems (Table 4). Importantly, our nomogram demonstrated excellent calibration, accurately estimating the probabilities of 28-day mortality in both cohorts when compared to the actual outcomes (Fig. 4; *P* > 0.05). These findings collectively affirm the superiority of our nomogram in predicting the likelihood of 28-day mortality when compared to widely used systems.

**Table 3:** Comparative assessment of C-index performance for nomogram and models incorporating disease severity scoring systems in predicting 28-day mortality among geriatric ischemic stroke patients.

Models	Development dataset		Validation dataset	
	C-index	95% CI	C-index	95% CI
Nomogram	0.809	0.778–0.841	0.786	0.737–0.835
SOFA	0.728	0.690–0.765	0.748	0.690–0.806
APSIII	0.712	0.674–0.749	0.712	0.653–0.771
LODS	0.708	0.671–0.745	0.729	0.673–0.786
SAPSII	0.744	0.709–0.779	0.766	0.715–0.818
OASIS	0.809	0.778–0.841	0.786	0.737–0.835

*C-index* concordance index, *CI* confidence interval, *SOFA* sequential organ failure assessment, *APS III* acute physiology score III, *LODS* logistic organ dysfunction system, *SAPS II* simplified acute physiology score II, *OASIS* oxford acute severity of illness score.

**Table 4:** A comparative evaluation of NRI and IDI in predictive models for 28-day mortality in geriatric individuals with ischemic stroke.

Index	Development dataset			Validation dataset		
	Estimate	95% CI	*P-*value	Estimate	95% CI	*P*-value
NRI (vs. SOFA)	0.304	0.225–0.384	< 0.001	0.333	0.218–0.448	< 0.001
NRI (vs. APS III)	0.205	0.123–0.286	< 0.001	0.107	0.038–0.251	0.148
NRI (vs. LODS)	0.244	0.162–0.327	< 0.001	0.257	0.135–0.379	< 0.001
NRI (vs. SAPS II)	0.275	0.193–0.357	< 0.001	0.190	0.047–0.333	0.009
NRI (vs. OASIS)	0.209	0.133–0.285	< 0.001	0.160	0.024–0.297	0.022
IDI (vs. SOFA)	0.201	0.169–0.232	< 0.001	0.164	0.114–0.213	< 0.001
IDI (vs. APS III)	0.126	0.096–0.157	< 0.001	0.067	0.013–0.121	0.016
IDI (vs. LODS)	0.146	0.115–0.177	< 0.001	0.121	0.071–0.171	< 0.001
IDI (vs. SAPS II)	0.155	0.121–0.188	< 0.001	0.094	0.039–0.149	0.001
IDI (vs. OASIS)	0.118	0.090–0.146	< 0.001	0.062	0.012–0.112	0.014

*NRI* net reclassification index, *IDI* integrated discrimination improvement, *CI* confidence interval, *SOFA* sequential organ failure assessment, *APS III* acute physiology score III, *LODS* logistic organ dysfunction system, *SAPS II* simplified acute physiology score II, *OASIS* Oxford acute severity of illness score.

Cutoff: 0, 0.4, 0.8, 1.

### Clinical value of the nomogram

**Figure 5:**
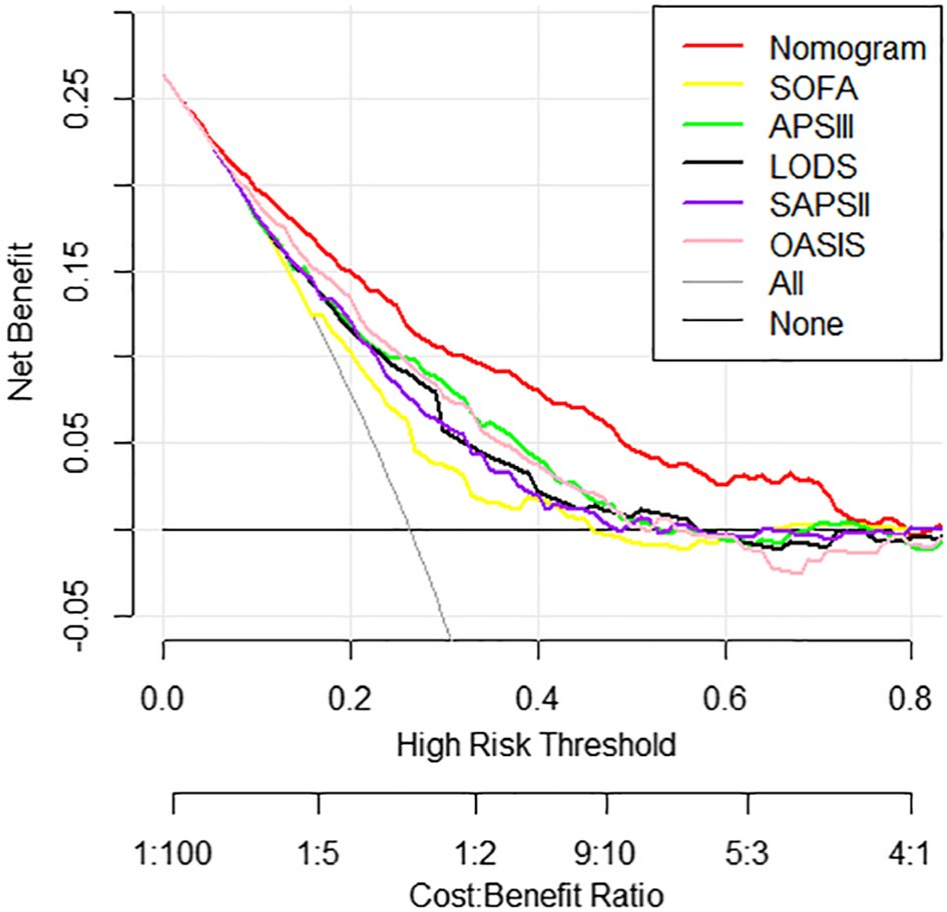
The decision curve analysis of the developed nomogram, along with models based on ICU severity scoring systems, was performed in the training set. The plotted results indicated that our nomogram exhibited the best performance, as represented by the red line, surpassing the other scoring systems. *SOFA* sequential organ failure assessment, *APS III* acute physiology score III, *LODS* logistic organ dysfunction system, *SAPS II* simplified acute physiology score II, *OASIS* oxford acute severity of illness score.

Subsequently, a comprehensive evaluation of clinical value was conducted using DCA in the training set. Remarkably, our constructed nomogram demonstrated superior net clinical benefits compared to the SOFA, APS III, LODS, SAPS II, and OASIS scoring systems. This enhanced clinical benefit of our nomogram is visually depicted by the red line in Fig. 5. Overall, our nomogram exhibited the most favorable performance, reaffirming its superiority in predicting 28-day mortality compared to the other scoring systems.

## Discussion

This study successfully identified and incorporated 9 independent variables associated with 28-day mortality in elderly individuals with ischemic stroke, leading to the development of a prognostic nomogram. The variables encompassed marital status, type of first care unit, presence of metastatic solid tumor, first-day urine output, platelet count, mannitol administration, heparin administration, mechanical ventilation, and minimum value of first-day GCS. Through comprehensive analyses including C-index, NRI, IDI, calibration curves, and DCA, our nomogram demonstrated satisfactory performance. Importantly, the nomogram outperformed scoring systems based on SOFA, APS III, LODS, SAPS II, and OASIS in terms of discrimination and net clinical benefits. These findings underscore the potential application of our constructed nomogram in clinical settings.

Previously, various prominent scoring systems, including SOFA, APS III, LODS, SAPS II, and OASIS, have been utilized to evaluate prognosis and severity with favorable outcomes^18–22^. However, their applicability in predicting outcomes specifically in elderly individuals with ischemic stroke remains uncertain. Notably, no dedicated scoring system has been developed thus far to assess the risk of 28-day mortality in this specific population. In contrast, the nomogram constructed in this study demonstrated superior performance in predicting short-term mortality, specifically within a 28-day timeframe, surpassing the predictive capabilities of the aforementioned commonly used clinical scoring systems.

The development of the SOFA score was initiated in 1994 as a standardized assessment tool encompassing six different scores^23^. Although the SOFA score has demonstrated efficacy in accurately predicting outcomes in severe acute ischemic stroke cases^24^, our study revealed its inferior performance in predicting short-term mortality specifically in elderly patients with ischemic stroke. This discrepancy can be attributed to the fact that the SOFA score primarily focuses on assessing organ function rather than predicting outcomes, with its application primarily directed towards sepsis patients rather than the elderly ischemic stroke population. The APS III score, derived from the physiology sub-score of acute physiology and chronic health evaluation (APACHE) III, was initially introduced by Knaus et al. in 1991^25^. This scoring system encompasses 14 indicators. In our study, utilizing the APS III scoring system for predicting 28-day mortality in elderly individuals with ischemic stroke yielded a C-index of 0.728. LODS, introduced by Le Gall et al. in 1996^26^, has demonstrated enhanced stability compared to APACHE II in previous prognostic studies involving neurological ICU patients^27^. However, our findings revealed the superior performance of our nomogram in comparison to the LODS score, which predominantly focuses on assessing the nervous system.

SAPS II, an improvement upon SAPS I introduced by Le Gall et al.^28^, has been further developed into SAPS III. However, studies have revealed that the SAPS III score tends to overestimate mortality in ICU patients with internal disorders when compared to the SAPS II score^29^. In contrast, our nomogram exhibited superior accuracy in predicting 28-day mortality among the elderly ischemic stroke population compared to SAPS II, which encompasses 15 variables. Notably, the application of complex scoring systems in clinical settings is often challenging due to their requirement for numerous indicators^30^. Thus far, limited studies have examined the utilization of OASIS, a machine-learning-based scoring system with a reduced number of factors^31^, specifically in the context of the neurological ICU population. Zhu et al.^22^ reported that OASIS displayed the highest predictive performance for mortality in critically ill individuals within the neurological domain. However, our current study demonstrated that OASIS exhibited inferior predictive capability in elderly ischemic stroke, ranking second only to our developed nomogram.

Our study revealed that elderly individuals who were widowed experienced poorer outcomes following an ischemic stroke, whereas those who were married exhibited a more favorable prognosis. Marriage plays a critical role in providing essential social support and facilitating access to stable behavioral and psychosocial resources^32^. Conversely, the experience of spousal loss can precipitate acute stress related to the process of bereavement, coupled with chronic stress arising from diminished emotional, financial, and social support^33^. Previous studies have indicated potential benefits of admission to specialized neurocritical care units for critically ill neurology patients, including reduced in-hospital mortality and shorter length of stay when managed by dedicated neurocritical care teams^34^. However, the establishment of specialized neurological care units does not appear to have a direct association with the mortality rate of acute ischemic stroke patients^35^. Similarly, our research findings suggest that the admission of elderly ischemic stroke patients to specialized Neuro ICU compared to medical or surgical ICUs does not significantly impact short-term mortality. These findings contribute to the growing body of evidence on the role of marital status and specialized care units in the context of stroke outcomes.

The presence of tumors bears a close association with the prognosis of ischemic stroke. Notably, approximately one out of every ten hospitalized patients with ischemic stroke presents with comorbid cancer^36^. Ischemic stroke patients with a recent history of cancer demonstrate a substantially elevated unadjusted incidence rate of all-cause mortality compared to those without a prior cancer diagnosis^37^. Similarly, we posit that elderly patients with ischemic stroke who have underlying metastatic solid tumors demonstrate a heightened risk of short-term mortality. The increased mortality observed in this subgroup can be attributed to the additional burden imposed by cancer, with hypercoagulability among individuals with active cancer serving as a significant contributing factor^38^. The GCS score serves as a widely employed, straightforward, and non-invasive clinical tool for assessing coma severity, which may in turn reflect the severity of stroke. Notably, a lower GCS score has demonstrated predictive value for hospital mortality^39^. Our study further reinforces the significance of GCS as a prognostic indicator for short-term mortality among elderly individuals with ischemic stroke.

The relationship between urine output and stroke remains relatively understudied. Our investigation reveals that lower first-day urine output serves as an independent risk factor for short-term mortality in elderly patients with ischemic stroke. It is noteworthy that decreased hourly urine output has been associated with moderate hypothermia in stroke patients devoid of concurrent cardiovascular or physiological disorders^40^. Additionally, a decline in urine output may partly signify a reduction in renal function. However, in our study, body temperature and blood creatinine levels were not found to be independent risk factors for short-term mortality in elderly ischemic stroke patients. Additionally, our study has revealed that a higher platelet count serves as an independent risk factor for 28-day mortality among elderly patients with ischemic stroke. Moreover, elevated platelet levels have been associated with an increased risk of recurrent stroke and poorer functional outcomes^41^. The underlying rationale for these observations lies in the fact that platelet aggregation-related thrombosis represents a critical step in the pathophysiology of ischemic stroke^42^. As a result, intervention studies have supported the implementation of antiplatelet therapy in specific populations with ischemic stroke, such as administering antiplatelet therapy before mechanical thrombectomy, which may indirectly improve the surgical success^43^.

In the current study, several interventions, including mannitol administration, heparin administration, and MV, were identified as significant independent prognostic indicators for predicting short-term mortality among elderly ischemic stroke patients. While there are recommendations supporting the use of osmotic therapy, including mannitol, for patients with clinically worsening cerebral edema caused by ischemic stroke^44^, our findings suggest caution. Specifically, in patients with cerebral edema caused by acute ischemic stroke, mannitol administration appears to be associated with an increased risk of death, independent of the stroke severity^45^. This aligns with existing perspectives that raise concerns about the use of mannitol in this patient population. It is essential to note that the efficacy of mannitol relies on the integrity of the blood–brain barrier, as it functions to facilitate dehydration and lower intracranial pressure by creating an osmotic gradient between cerebral vessels and brain parenchyma^46^. However, in the unfortunate circumstance of a compromised blood–brain barrier, mannitol administration may lead to the transfer of osmotic substances to brain tissue, aggravating edema due to the reversal of the osmotic gradient^46^. This adverse effect underscores the potential complexities and risks associated with mannitol use in the context of ischemic stroke. Hence, the utilization of intravenous penetrants as a preventive measure for short-term mortality in elderly patients with acute ischemic stroke is not supported^47^. In a word, our study adds to the growing body of evidence supporting the critical consideration of mannitol administration as an independent risk factor for 28-day mortality in elderly patients with ischemic stroke. Further research and clinical vigilance are warranted to optimize treatment strategies and mitigate potential adverse outcomes in this vulnerable population.

This study highlights that the administration of heparin is identified as an independent risk factor for short-term mortality in elderly patients with ischemic stroke. In recent years, a systematic review has indicated that although anticoagulation treatment for ischemic stroke patients can reduce the occurrence of recurrent stroke, deep vein thrombosis, and pulmonary embolism, it is also associated with an increased risk of bleeding^48^. However, early anticoagulation therapy does not appear to have a significant impact on short-term mortality in patients with acute ischemic stroke^48^. Therefore, based on the current data, the routine use of anticoagulant therapy for acute ischemic stroke is not supported^48^.

Our previous research has corroborated that MV is an independent risk factor for long-term mortality in patients with ischemic stroke^6^. In this current study, we further ascertain that MV also constitutes an independent risk factor for short-term mortality in elderly patients with ischemic stroke. Remarkably, the 30-day mortality rate for acute ischemic stroke patients necessitating invasive mechanical ventilation reaches as high as 56%^49^. In-depth investigations have delved into the clinical implications of MV duration on ischemic stroke patients, revealing that those undergoing mechanical thrombectomy experienced worse 3-month outcomes when the ventilation time exceeded 24 h^50^. The requirement for MV serves as an indicator of the critical condition of acute stroke patients^51^. Moreover, prolonged MV has been associated with an increased incidence of complications, such as pneumonia, and is linked to unfavorable prognosis^50^.

This study is subject to several limitations that warrant consideration. Firstly, being a retrospective study utilizing the MIMIC-IV database, it does not allow for the establishment of definitive causal relationships between risk factors and outcome indicators. As such, we advocate for the need for further investigation through Mendelian randomization randomized studies and prospective cohort studies to explore these potential causal relationships more comprehensively. Secondly, the limitations of the MIMIC database also mean that certain crucial variables, including neuroimaging and electrophysiological examinations, among others, were not incorporated into this study. In future database construction endeavors, we recommend the inclusion of detailed specialized materials to enhance the scope of analysis. Thirdly, it is important to note that our validation efforts were limited to internal validation only. To ensure the robustness and generalizability of the established nomogram, additional high-quality research and studies using external validation and test sets are imperative. These measures will help validate and strengthen the reliability of our nomogram's predictive performance in elderly ischemic stroke patients.

## Conclusion

In conclusion, our findings revealed significant associations between 28-day mortality and various factors, such as marital status, type of first care unit, presence of metastatic solid tumor, first-day urine output, platelet count, mannitol administration, heparin administration, MV, and minimum value of first-day GCS in elderly ischemic stroke patients. Leveraging LASSO and multiple logistic regression algorithms, we successfully developed and validated a nomogram capable of accurately predicting short-term mortality (28-day) in this patient population. Our nomogram demonstrated favorable discrimination, calibration, and net clinical benefits, underscoring its potential for evaluating patient mortality and enhancing medical condition communication in clinical practice. To further ascertain the nomogram's performance, external validation and test sets are warranted in future studies. Such efforts will contribute to validating and solidifying the reliability of our nomogram in assessing short-term mortality risk in elderly ischemic stroke patients.

## Data Availability

Raw data for this study are available at: https://physionet.org/content/mimiciv/2.1/.

## Abbreviations

ICU: Intensive care unit
IQR: Interquartile range
GCS: Glasgow coma score
APS III: Acute physiology score III
SOFA: Sequential organ failure assessment
LODS: Logistic organ dysfunction system
SAPS II: Simplified acute physiology score II
OASIS: Oxford acute severity of illness score
MICU: Medical Intensive Care Unit
SICU: Surgical Intensive Care Unit
Neuro SICU: Neuro Surgical Intensive Care Unit
TSICU: Trauma Surgical Intensive Care Unit
APACHE: Acute physiology and chronic health evaluation
LOS: Length of stay
OR: Odd ratio
CI: Confidence interval
C-index: Concordance index
NRI: Net reclassification index
IDI: Integrated discrimination improvement
DCA: Decision curves analysis
LASSO: Least absolute shrinkage and selection operator
MIMIC-IV: Medical information mart for intensive care IV
MV: Mechanical ventilation
BIDMC: Beth Israel Deaconess Medical Center
MIT: Massachusetts Institute of Technology
ICD: International classification of disease
CCI: Charlson comorbidity index
OR: Odd ratio
CI: Confidence interval

## References

[1] Collaborators GBDS (2021). Global, regional, and national burden of stroke and its risk factors, 1990–2019: A systematic analysis for the Global Burden of Disease Study 2019. Lancet Neurol..

[2] Kavga A (2021). The effects of patients' and caregivers' characteristics on the burden of families caring for stroke survivors. Int. J. Environ. Res. Public Health.

[3] Katan M, Luft A (2018). Global burden of stroke. Semin. Neurol..

[4] Global, regional, and national age-sex specific mortality for 264 causes of death, 1980–2016: A systematic analysis for the Global Burden of Disease Study 2016. *Lancet* **390**, 1151–1210 (2017). 10.1016/S0140-6736(17)32152-9 PMC5605883 28919116

[5] Marini C (2004). Burden of first-ever ischemic stroke in the oldest old: Evidence from a population-based study. Neurology.

[6] Jin G, Hu W, Zeng L, Ma B, Zhou M (2023). Prediction of long-term mortality in patients with ischemic stroke based on clinical characteristics on the first day of ICU admission: An easy-to-use nomogram. Front. Neurol..

[7] Deng QW (2018). The short-term prognostic value of the triglyceride-to-high-density lipoprotein cholesterol ratio in acute ischemic stroke. Aging Dis..

[8] Yu Q (2021). Development and internal validation of a multivariable prediction model for 6-year risk of stroke: A cohort study in middle-aged and elderly Chinese population. BMJ Open.

[9] Meng L (2020). Nomogram to predict poor outcome after mechanical thrombectomy at older age and histological analysis of thrombus composition. Oxid. Med. Cell Longev..

[10] Yang Y (2020). Development of a nomogram to predict 30-day mortality of patients with sepsis-associated encephalopathy: A retrospective cohort study. J. Intensive Care.

[11] Johnson, A. *et al.* MIMIC-IV (version 2.1). *PhysioNet* (2022).

[12] Cai G (2022). Optimal targets of the first 24-h partial pressure of carbon dioxide in patients with cerebral injury: Data from the MIMIC-III and IV database. Neurocrit. Care.

[13] Charlson M, Szatrowski TP, Peterson J, Gold J (1994). Validation of a combined comorbidity index. J. Clin. Epidemiol..

[14] Quan H (2005). Coding algorithms for defining comorbidities in ICD-9-CM and ICD-10 administrative data. Med. Care.

[15] Guo X (2021). Development and validation of survival nomograms in colorectal cancer patients with synchronous liver metastases underwent simultaneous surgical treatment of primary and metastatic lesions. Am. J. Cancer Res..

[16] Wu J (2020). A nomogram for predicting overall survival in patients with low-grade endometrial stromal sarcoma: A population-based analysis. Cancer Commun. (Lond.).

[17] Zhou ZR (2019). In-depth mining of clinical data: The construction of clinical prediction model with R. Ann. Transl. Med..

[18] Basile-Filho A (2019). The use of APACHE II, SOFA, SAPS 3, C-reactive protein/albumin ratio, and lactate to predict mortality of surgical critically ill patients: A retrospective cohort study. Medicine (Baltimore).

[19] Pang K, Li L, Ouyang W, Liu X, Tang Y (2022). Establishment of ICU mortality risk prediction models with machine learning algorithm using MIMIC-IV database. Diagnostics (Basel).

[20] Sarkar R (2021). Performance of intensive care unit severity scoring systems across different ethnicities in the USA: A retrospective observational study. Lancet Digit Health.

[21] Kara H (2015). Red cell distribution width and neurological scoring systems in acute stroke patients. Neuropsychiatr. Dis. Treat..

[22] Zhu S (2022). Predictive value of six critical illness scores for 28-day death risk in comprehensive and specialized intensive care unit patients based on MIMIC-IV database. Zhonghua Wei Zhong Bing Ji Jiu Yi Xue.

[23] Vincent JL (1996). The SOFA (Sepsis-related Organ Failure Assessment) score to describe organ dysfunction/failure. On behalf of the Working Group on Sepsis-Related Problems of the European Society of Intensive Care Medicine. Intensive Care Med..

[24] Qin W (2020). Predictive value of the sequential organ failure assessment (SOFA) score for prognosis in patients with severe acute ischemic stroke: A retrospective study. J. Int. Med. Res..

[25] Knaus WA (1991). The APACHE III prognostic system. Risk prediction of hospital mortality for critically ill hospitalized adults. Chest.

[26] Le Gall JR (1996). The Logistic Organ Dysfunction system. A new way to assess organ dysfunction in the intensive care unit. ICU Scoring Group. Jama.

[27] Kim TK, Yoon JR (2012). Comparison of the predictive power of the LODS and APACHE II scoring systems in a neurological intensive care unit. J. Int. Med. Res.

[28] Le Gall JR, Lemeshow S, Saulnier F (1993). A new Simplified Acute Physiology Score (SAPS II) based on a European/North American multicenter study. JAMA.

[29] Brusca RM (2020). Performance of critical care outcome prediction models in an intermediate care unit. J. Intensive Care Med..

[30] Wang L, Zhang Z, Hu T (2021). Effectiveness of LODS, OASIS, and SAPS II to predict in-hospital mortality for intensive care patients with ST elevation myocardial infarction. Sci. Rep..

[31] Johnson AE, Kramer AA, Clifford GD (2013). A new severity of illness scale using a subset of Acute Physiology And Chronic Health Evaluation data elements shows comparable predictive accuracy. Crit. Care Med..

[32] Reeves MJ, Prager M, Fang J, Stamplecoski M, Kapral MK (2014). Impact of living alone on the care and outcomes of patients with acute stroke. Stroke.

[33] Dong L, Briceno E, Morgenstern LB, Lisabeth LD (2020). Poststroke cognitive outcomes: Sex differences and contributing factors. J. Am. Heart Assoc..

[34] Suarez JI (2004). Length of stay and mortality in neurocritically ill patients: Impact of a specialized neurocritical care team. Crit. Care Med..

[35] Bershad EM, Feen ES, Hernandez OH, Suri MF, Suarez JI (2008). Impact of a specialized neurointensive care team on outcomes of critically ill acute ischemic stroke patients. Neurocrit. Care.

[36] Sanossian N, Djabiras C, Mack WJ, Ovbiagele B (2013). Trends in cancer diagnoses among inpatients hospitalized with stroke. J. Stroke Cerebrovasc. Dis..

[37] Akyea RK (2023). Cardiovascular outcomes and mortality after incident ischaemic stroke in patients with a recent cancer history. Eur. J. Intern. Med..

[38] Lee MJ (2017). Hypercoagulability and mortality of patients with stroke and active cancer: The OASIS-CANCER study. J. Stroke.

[39] Shah B (2017). Predictors of in-hospital mortality of acute ischemic stroke in adult population. J. Neurosci. Rural Pract..

[40] Guluma KZ (2010). Therapeutic hypothermia is associated with a decrease in urine output in acute stroke patients. Resuscitation.

[41] Yang M (2019). Platelet count predicts adverse clinical outcomes after ischemic stroke or TIA: Subgroup analysis of CNSR II. Front. Neurol..

[42] Kraft P, Schwarz T, Meijers JC, Stoll G, Kleinschnitz C (2010). Thrombin-activatable fibrinolysis inhibitor (TAFI) deficient mice are susceptible to intracerebral thrombosis and ischemic stroke. PLoS ONE.

[43] Çabalar M, Şengeze N, Eren A, Inanç Y, Giray S (2022). How does the use of antiplatelet and anticoagulants affect the success of mechanical thrombectomy in acute ischemic stroke cases?. Ideggyogy Sz..

[44] Wijdicks EF (2014). Recommendations for the management of cerebral and cerebellar infarction with swelling: A statement for healthcare professionals from the American Heart Association/American Stroke Association. Stroke.

[45] Papagianni M (2018). Treatment with mannitol is associated with increased risk for in-hospital mortality in patients with acute ischemic stroke and cerebral edema. Am. J. Cardiovasc. Drugs.

[46] Diringer MN, Zazulia AR (2004). Osmotic therapy: Fact and fiction. Neurocrit. Care.

[47] Zuliani G (2004). Prescription of anti-oedema agents and short-term mortality in older patients with acute ischaemic stroke. Drugs Aging.

[48] Wang X (2021). Anticoagulants for acute ischaemic stroke. Cochrane Database Syst. Rev..

[49] de Montmollin E (2019). Pneumonia in acute ischemic stroke patients requiring invasive ventilation: Impact on short and long-term outcomes. J. Infect..

[50] Fandler-Höfler S (2020). Ventilation time and prognosis after stroke thrombectomy: The shorter, the better!. Eur. J. Neurol..

[51] Horner RD, Sloane RJ, Kahn KL (1998). Is use of mechanical ventilation a reasonable proxy indicator for coma among Medicare patients hospitalized for acute stroke?. Health Serv. Res..

